# Time Spent at Work and Its Impact on the Academic Performance of Pharmacy Students

**DOI:** 10.3390/ijerph17020496

**Published:** 2020-01-13

**Authors:** John Okogbaa, Rondall E. Allen, Daniel F. Sarpong

**Affiliations:** 1 Division of Clinical and Administrative Sciences, Xavier University of Louisiana College of Pharmacy, New Orleans, LA 70125, USA; jokogbaa@xula.edu; 2 Pharmacy Practice and Administration, University of Maryland Eastern Shore School of Pharmacy and Health Professions, Princess Anne, MD 21853, USA; reallen@umes.edu

**Keywords:** pharmacy students, workload, academic performance, grade point average, professional years

## Abstract

The objective of this study was to determine the impact of time spent at work (workload) on the academic performance of pharmacy students. A cross-sectional 12-item survey was administered to pharmacy students at the end of the spring 2011 semester to primarily assess the type of employment and their weekly workload during the 2010–2011 academic year. Academic performance was determined by semester and cumulative grade point average (GPA). Descriptive statistics were performed. Stratified multiple linear regression models were obtained to assess the association between students’ workload and GPA. Analysis of covariance was used to compare academic performance by workload after accounting for work type and potential covariates. Statistical significance was defined a priori as *p* < 0.05. For both fall and spring semesters, nonpharmacy-related work was significant and positively associated with GPA. Both semester GPAs were fairly similar among three student classifications (P1–P3). However, GPAs across both semesters varied by classification. The negative association of workload on GPA was significant in the fall but not in the spring semester. Although workload matters, future studies using a mixed-method approach might help explain the role of workload on the academic performance of pharmacy during the first three years of their professional training.

## 1. Introduction

Several variables have been used to predict the success or failure of pharmacy students throughout the doctor of pharmacy (PharmD) curriculum; most notable has been the passing of the North American Pharmacist Licensure Examination (NAPLEX) test [1,2,3]. However, none of these studies have specifically looked at the impact of workload and the type of work as a predictor of success or failure in the PharmD curriculum. A meta-analysis by Kuncel et al. showed that prepharmacy grade point average (GPA) and Pharmacy College Admission Test (PCAT) score were predictors of success in the PharmD curriculum and on the national pharmacy board examination but did not consider the impact of nonacademic variables [4]. Interestingly, a study by Sansgiry et al. analyzed nonacademic variables that may impact progression in the PharmD program, such as test competence, time management, strategic studying, and test anxiety. They concluded that test competency was the only significant determinate of students’ academic progression based on GPA [5]. Admission pathways into the PharmD program may be a determinate of students’ success in the PharmD curriculum. In a study conducted by Schauner et al., admission pathways were classified as traditional or provisional [6]. The traditional pathway is the “1 plus 5” program: 1 year of prerequisite coursework is required before completing 5 years of the pharmacy curriculum. A minimum cumulative GPA of 2.75 or higher and a minimum science/math GPA of 2.5 or higher is required to be admitted under the traditional pathway, while the provisional admission was for high school seniors with a cumulative GPA of 3.6 and a minimum American College Testing (ACT) or Scholastic Aptitude Test (SAT) composite score of 23 or 1060, respectively. The provisionally admitted students were admitted to the five-year program based upon their completion of one year of prerequisite coursework and their performance on the PCAT [6]. It was inferred that students admitted to the program through the provisional pathway level struggled academically compared with those who entered through the traditional pathway [6]. The type of undergraduate degree earned or advanced course work prior to entering into PharmD program has also been shown to influence academic performance in the PharmD program. Students who earned a bachelor of science degree or advanced coursework in biology prior to entering pharmacy school were more likely to be more successful in the PharmD program compared with those with a bachelor of arts degree or any other degree. However, on a multivariable analysis, only advanced biology coursework remained a significant predictor of success [7].

An analysis of the impact of previous pharmacy-related work experiences on student performance in the college of pharmacy failed to find any significant relationship between number of hours worked or work setting and academic performance in the PharmD program [8]. The study suggested that type of work prior to PharmD program, be it retail pharmacy, hospital pharmacy, or community pharmacy, had a minimal effect on students’ performance while in pharmacy school [8]. A study by Charupatanapong et al. examined the relationship between the academic performance of pharmacy students and the number of hours worked; using a correlation matrix analysis, they found that there was an inverse relationship between GPA and hours worked. The study also found a positive correlation with prepharmacy GPA and study hours, however a negative correlation was found between GPA, working hours, and hours spent on extracurricular activities. They further concluded that no single set of variables reliably determines students’ progression in the college of pharmacy. The study also indicated that those with lower academic performance (GPA < 3.0) were likely to be those with less study time, more work hours, and high extracurricular activities compared with those with higher academic performance (GPA 3.0–4.0), showing a positive correlation [9].

Although no studies were found on the association of leadership and other related factors with the academic performance of pharmacy students, studies have documented that these nonacademic factors impact the performance of other health professional students. Studies have documented that nonacademic factors such as leadership/decisiveness, expected difficulty, and motivation have been used as a determinant of higher score in the United States Medical Licensure Exam—Step 1. In one of the two medical schools analyzed, it was found that these nonacademic variables were consistently related to student progression or performance, with leadership/decisiveness contributing the most [10]. A study published in Nurse Education Today estimated that nursing students who worked more than 16 h/week had lower academic performance compared with students who worked less than 16 h/week. The study also cited that longer working hours affected the students’ college life activity [11]. Contrary to this finding is a study by Nonis and Hudson, which concluded that the time spent working was not directly related to students’ academic performance [12].

The academic pharmacy literature is replete with academic variables or determinants of student academic progression in pharmacy school, and there is a paucity of articles that have been published on the impact of nonacademic variables. We found it important to examine the impact of time spent at work on the academic performance of pharmacy students and to determine if there is an association between the amount of hours spent at work and students’ GPA. With this purpose in mind, we believe that the outcome of this study will help college administrators in advising students on workload and progression in the colleges of pharmacy. To our knowledge, little or no studies have evaluated the relationship between the workload and academic performance of pharmacy students. We hypothesized that workload is negatively associated with academic performance.

## 2. Materials and Methods

### 2.1. Study Design

A cross-sectional survey methodology was utilized in the performance of this study.

### 2.2. Target Population and Study Sample Recruitment

The study population was pharmacy students in the first three years of their professional training (P1–P3) at Xavier University of Louisiana. Students in their professional year 4 (P4) were excluded because the primary outcome (GPA) does not change from the P3 to P4 years. A sample of 384 distinct students out of a total of 474 students was recruited for this study. Flyers were posted on notice boards and email communications were sent to students to complete the survey part of the study. Academic performance data were obtained from the academic records of consenting students enrolled in the study.

### 2.3. Measurement and Data Collection

A 12-item survey instrument was developed to assess the association between time spent at work and the academic performance of pharmacy students. The survey was divided into three sections. The first section contained eight items of demographic data such as name, student identification number, class, gender, ethnicity, marital status, age, and number of children. The students’ name and identification number were collected in order to obtain their GPA and academic standing at the end of each semester. To ensure confidentiality, student names and identification numbers were stripped from the final analytic dataset. The second and third sections contained two items each and focused on the type of work and the number of hours worked per week during the fall 2010 and spring 2011 semesters, independently.

Using the Delphi method, a review of the survey items was conducted by a group of students in the College of Pharmacy and three faculty members who are well versed in assessment and survey research. The students were asked to review the instrument to ensure the instructions were clear, to identify ambiguous questions, and to provide comments on the format of the survey tool. The faculty members evaluated content validity. The survey instrument was modified based upon feedback from both groups.

Upon approval by the Institutional Review Board at Xavier University of Louisiana, the survey was administered to all of the first-, second-, and third-year students enrolled in the College of Pharmacy during the spring 2011 semester. A course in each professional year was identified and used to administer the survey at the end of the class. A consent statement was read before the survey was given. Consenting to participate in the study equated with completing the survey. The incentive for study participation was a gift card. The survey was also administered one week later to ensure that students who did not participate in the survey the week prior due to missing class or other reasons were afforded the opportunity to participate. The researchers reviewed all of the surveys that were submitted by the students to ensure all of the questions were answered. Students who submitted surveys with missing information were contacted immediately by one of the researchers to ensure that missing data due to omission were addressed by the student.

### 2.4. Pharmacy Curriculum

**Figure 1:**
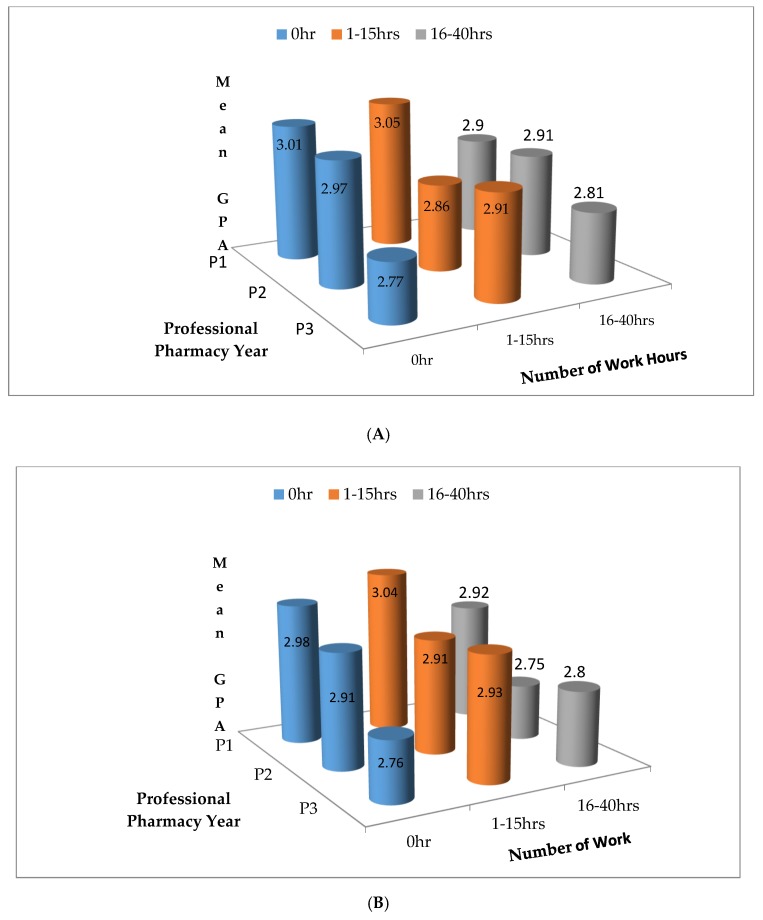
(**A**) Adjusted means of fall Grade Point Average (GPA) of study relative to workload by professional pharmacy year. (**B**) Adjusted means of spring GPA of study relative to workload by professional pharmacy year. hrs = hours.

The Xavier University of Louisiana College of Pharmacy (XULA COP) is a four-year curriculum with a total of 104 credit hours (chrs). The first three professional years (P1–P3) are based on didactic training and the fourth professional year (P4) is experiential training involving seven six-week rotations. The P1 and P2 years (35 chrs each year) cover introduction to pharmacy/healthcare and primarily basic science courses foundational to pharmacy and most medical and allied health sciences. The P1 and P2 courses are focused on the basic sciences, biological, and physical sciences, whereas the P3 courses are mainly clinical science and practice-related courses. The PharmD curriculum is 102 credit hours of didactic courses and 48 experiential hours. The didactic courses are offered in the first three years (P1–P3) of the program. In the professional pharmacy year one (P1), there are a total of 33 didactic hours, which cover introductory courses in pharmacy and basic courses that are a continuation of the biological and physical sciences courses offered in undergraduate science programs. For the fall and spring semesters, 22 out of the 33 credit hours are generally considered the challenging courses. These challenging courses as flagged in Figure 1 by an asterisk. The second year (P2) is considered the toughest of the first three years of the program, in that the students transition into graduate-level basic science courses and are introduced to clinical science courses required in disease management. In the P2 year, 29 out of 35 credit hours for the fall and spring semesters are considered challenging, which are flagged by an asterisk in Figure 1. In the third year (P3), the students focus on pharmacy courses that entail clinical, pharmacy management, law, and research methods to equip them for the practice of pharmacy. About 14 out of the 34 credit hours at this stage of the training might be considered challenging and are flagged by an asterisk in Figure 1. Hence, one can infer that the most challenging year for a pharmacy student is the second year (P2). Table 1 provides a summary of the pharmacy curriculum for 2010–2011.

### 2.5. Outcome Measure

The principal outcome measure for this study was GPA for both fall 2010 and spring 2011 semesters. Another potential outcome measure was academic standing, which includes good standing, probation, and dismissal. However, this measure is not reported because only 2.9% were not of good standing for both the fall 2010 and spring 2011 semesters. A student is in “good standing” unless on academic probation or academically dismissed. Students on academic probation have either a cumulative or semester GPA of less than 2.0 in the fall or spring semester or two or more grades of “D” or a grade of “F” in any semester. Students will be dismissed from the College of Pharmacy if they earn three or more grades of “F”, qualify for probation while already on probation, are placed on probation three times prior to their senior year, fail to pass a course on the second attempt, are ineligible to begin their senior rotations at the end of eight regular semesters, and earn more than a grade of “F” during their senior year.

### 2.6. Independent Measures

Type of work and number of work hours (workload) in the fall 2010 and spring 2011 semesters were the principal independent variables. Type of work which was classified as none (not employed), pharmacy-related (chain retail pharmacy, independent retail pharmacy, hospital retail pharmacy, and hospital pharmacy), and nonpharmacy-related work, which were used to derive two dummy variables: pharmacy related and nonpharmacy related. Hence, for data analysis, the use of these two dummy variables implies the comparison of pharmacy-related work versus no employment and nonpharmacy-related work versus no employment. Work hours for both fall 2010 and spring 2011 were captured on the survey as 0–10, 11–20, 21–30, 31–40, and >40 h per week. To treat it as a continuous measure, the variable was transformed into 0 if a student was not employed and 10, 15, 25, 35, and 40 h per week if they selected 0–10, 11–20, 21–30, 31–40, and >40 h, respectively.

### 2.7. Covariates

The following variables were treated as covariates and adjusted for in the data analysis: student classification (P1–P3), number of Pharmacy Board hours completed out the 500 required, and demographic factors (gender, age, marital status, number of children, and race/ethnicity). Involvement in student organizations and fraternities or sororities was also considered.

An index for involvement in student organizations was derived using the following four items: membership in a professional student organization, number of organizations holding membership, level of involvement in terms of holding position, and duration of membership. Similarly, an index for involvement of fraternities or sororities was derived using the following three items: membership in a fraternity or sorority, level of involvement in terms of holding position, and duration of membership.

### 2.8. Data Analytic Plan

Descriptive statistics were used to describe the characteristics of the study sample. Two sets of four multiple linear regression models were obtained to assess the association between the number of hours worked by pharmacy students and their GPA for the fall and spring semesters. These regression models were stratified by semester (fall 2010 and spring 2011). Model I examined the relationship between the number of hours worked and type of work with student classification and number of pharmacy board hours completed as covariates. Model II was Model I and the demographic factors (gender, age, marital status, number of children, and race/ethnicity). Model III was Model II and involvement in student professional organizations. Model IV was Model III and involvement in fraternities or sororities (fully adjusted model). The four-staged regression modeling approach was used to determining the mediating role of the additional factors on the association between the number of hours worked by pharmacy students and their GPA, as these factors were included in the models. Additional analysis of covariance (ANCOVA) was used to compare academic performance by type of work after accounting for the numbers of hours worked and all the covariates. All statistical analyses were performed using SAS software version 9.4 (SAS Institute Inc., Cary, NC, USA). Statistical significance was defined a priori as *p* < 0.05.

## 3. Results

The survey was completed by 384 (81%) of the total population of P1–P3 students. Table 2 summarizes the characteristics of the study sample. The age distributions of the three classes (P1–P3) of pharmacy students were fairly comparable, with a mean (standard deviation) age of 24.8 (4.2) years and a range of 47 years. Females comprised 6%–70.8% of each of the classes, with the lowest and highest in the P3 and P2 years, respectively. The race/ethnicity of the students in the three classes were quite different. However, for all three classes, African Americans had the largest representation, ranging from 38.4% (P1) to 53.9% (P2). The number of children by the students were quite similar across the three classes. There was an obvious inverse trend in not being employed from the P1 year to P3 year, with the larger percentage of those employed working at a chain store. Membership in and the number of pharmacy-related student organizations increased with classification (P1–P3). The P2 students had the greatest percentage of memberships in nonpharmacy organizations (13.9%) compared with the P1s (7.5%) and the P3s (5.8%). However, the P3s had more officers in nonpharmacy organizations (4.1%) compared with the P1s (0.0%) and P2s (1.5%). The fall and spring semester GPAs were fairly similar among the three classifications.

Figure 1A,B provide a graphical depiction of the unadjusted relation between hours worked (workload) and GPA for the fall and spring semesters, respectively. Statistically, there were not significant differences in GPA for the three classes of workload within each of the three student classifications (P1–P3). However, the relationship between workload and GPA by classification differed between the fall and spring semesters. For both the fall and spring semesters, the P1 students who worked 1–15 h weekly had the highest GPA, followed by those who did not work (0 h). However, for the P2s, the distribution of GPA by workload was different between the fall and spring semesters. Similar to the P1s, for both the fall and spring semesters, the P3 students who worked 1–15 h weekly had the highest GPA, followed by those who did not work (0 h).

Table 3 summarizes the association between workload and academic performance measured by GPA for the fall and spring semesters. For both the fall and spring semesters, pharmacy-related work compared to not being employed was not significantly related to GPA. However, nonpharmacy-related work was significantly and positively associated with GPA, from the unadjusted model (Model I) to the fully adjusted model (Model IV). There were differences in the significance of the association of workload and GPA (academic performance) by semester. For the fall semester, workload was negatively associated with GPA. This was through Model I to the fully adjusted model (Model IV). However, for the spring semester, the negative association between workload and GPA was attenuated (marginally) for Models III and IV.

## 4. Discussion

The involvement of pharmacy students in professional pharmacy organizations increased as they matriculated through the program. The GPA varied with the number of hours a student worked and his/her classification (P1–P3). It might be that at the different stages of a student’s education, the type of work might be different. It is more likely that a P3 might be working in a pharmacy-related job than a P1, who has limited training in pharmacy.

Similar to the findings of Mar et al., for the fall semester, pharmacy-related work was not a correlate to academic performance measured by GPA. However, nonpharmacy-related work was positively correlated with academic performance. This pattern of relationship between academic performance (GPA) was also described by Rochford et al., who found that students who worked less than 16 h per week had better academic performance than students who worked more than 16 h [11]. Additionally, the number of hours a pharmacy student worked per week was negatively correlated with academic performance. This finding suggests that as work hours per week increased, it had a negative impact on students’ academic performance. Based on the data displayed in Figure 1, one could infer that an appreciable workload might be 1–15 h per week. However, beyond 15 h per week, for the derived benefit of working, the law of diminishing returns on academic performance sets in for the pharmacy student. It is important to note that at the time that this survey was conducted, the Louisiana Board of Pharmacy was still requiring students to complete 500 intern hours; this requirement may have put an additional demand on students to work more hours, especially in the third year.

The noted difference in the students’ GPA in the first three professional years and the difference between the fall and spring semesters might be explained by the following factors: challenging courses; fall performance might motivate students to study harder in the spring or cause study to relax; an ideal amount of work might result in better time management compared with zero hours of work, or too many hours of work (≥16 h) might have a negative impact. Also, it is important to note that in the P1 and P2 years, traditionally challenging courses such as Disease State Management I–III, Applied Pharmacokinetics, Medicinal Chemistry I and II, and Pharmacology I and II are offered. On the subject of an ideal workload possibly resulting in better time management compared with zero hours of work or a heavy workload (≥16 h), the data suggest that working just 1–15 h per week may have some academic benefit for P3 students. In the fall semester, the mean GPA for those working zero hours was 2.77, while for those working between 1–15 h, the mean GPA increased to 2.91; however, the GPA fell as work hours increased to 16–40. A similar relationship was also observed in the in the spring semester; mean GPA for zero work hours was 2.75 but increased to 2.93 as work hours increased to 1–15 h; however, mean GPA fell to 2.80 as work hours increased to 16–40.

The trends of GPA for both the fall and spring semesters were similar. For both P1 and P3 students, the distribution was an inverted U shape, and for P2 students, it was flat for 0 and 1–15 h of work and there was a decline in the 16–40 h a week category. For both semesters and all three classifications of pharmacy students, the data suggest that working 16–40 h negatively impacted the academic performance of the students. Also, the regression analysis for both semesters confirmed that there is a significantly inverse relationship between workload academic performance measured by GPA by classification (P1–P3). This finding is supported by a study by García-Vargas et al., who found a negative impact on academic performance of nursing students in Colombia, particularly if the students worked more than 20 h per week [13]. Studies by Salamonson and Andrew and Reyes et al. documented that students working more than 16 h per week experienced negative impacts on academic performance [14,15]. One important factor when examining the impact of workload on GPA of pharmacy students is that studies have shown that GPA is a predictor of the passing of the NAPLEX test [16]. Although changes in content delivery might influence GPA and performance on the NAPLEX, in this study, the delivery of the curriculum was fairly consistent, given that the same set of faculty members taught the courses to the set of students who served as study participants. Hence, course delivery is an unlikely confounder for which adjustment would be necessary.

However, for the spring semester, only nonpharmacy-related work was a positive correlate to academic performance. There was no significant association of a pharmacy student’s workload and academic performance. However, the association, which is negative, is in the right direction. Mar et al. in their study found that work type prior to the PharmD program, be it retail pharmacy, hospital pharmacy, or community pharmacy, had a very minimal effect on students’ performance while in pharmacy school [8]. Based on this study data and the findings of Mar et al., one might hypothesize that work type during the PharmD program might not have much of an impact on pharmacy students’ academic performance.

### Strengths and Limitations

The strengths of this study are threefold. First, the response rate of the survey was 81%. Second, the information, although collected in the 2010–2011 academic year, is still relevant in that the PharmD curriculum has not changed much; changes have been on content delivery. Third, findings from this cross-sectional study provide insights into examining the impact of workload on academic performance of pharmacy and other health professional students using a cohort-based study design, which is a design that requires more time and resources to conduct. The limitations of this study are that the design is cross sectional and, therefore, causality cannot be inferred. Second, the data might seem dated and might need to be replicated with more current data under the current curriculum which was implemented four years ago. Lastly, using a mixed method approach could better explain some of the context related to how working impacted students’ studies, which was not ascertained in this study.

## 5. Conclusions

The fall and spring semester GPAs were fairly similar among the three classifications. However, the distribution of GPAs across the fall and spring semesters varied by classification (P1–P3). Although the negative association of workload on GPA was significant in the fall, it was marginally significant in the spring semester after accounting for potential covariates. Given that workload does not have a linear effect, it would be prudent to examine this in a future study by means of a qualitative study or using a mixed method approach. The quantitative study aspect of the mixed method approach will enable researchers to quantify the effect of the impact of workload on academic performance, while the qualitative student component might explain the role of workload in the academic performance of pharmacy students through their first three years of professional training.

## Figures and Tables

**Table 1:** Doctor of pharmacy (PharmD) curriculum 2010–2011.

Fall Semester Course Offerings			Spring Semester Course Offerings	
		chr		chr
**P1**	Biomedical Immunology Human Anatomy and Physiology * Human Anatomy Lab Introduction to Pharmacy Pharmacy Calculation * Biostatistics Pharmaceutical Biochemistry * Introductory Pharmacy Practice Experience—I (IPPE-I) Introductory Pharmacy Practice Experience—I Seminar	2 3 1 2 2 3 3 1 0	Human Anatomy * Pharmacy Skills Lab—I Pharmaceutics—I * Principles of Drug Action * Molecular Biology and Biotechnology * Introductory Pharmacy Practice Experience—I (IPPE—I) Pharmacy Elective Introductory Pharmacy Practice Experience—I Seminar	3 1 4 2 3 1 2 (3) 0
	TOTAL	17	TOTAL	16 (17)
**P2**	Medicinal Chemistry—I * Pathophysiology * Pharmacology—I Pharmaceutics—II * Pharmacy Skills Lab—II Pharmaceutical Sciences lab Introductory Pharmacy Practice Experience—II (IPPE—II)	3 4 4 3 1 1 1	Disease State Management (DSM)—I * Behavioral Pharmacy and Communication Medicinal Chemistry—II * Pharmacology—II * Biopharmaceutics and Basic Pharmacokinetics * DSM—I Drill/Case Studies Behavioral Pharmacy and Communication Drill Introductory Pharmacy Practice Experience—II (IPPE—II)	5 2 3 4 3 0 0 1
	TOTAL	17	TOTAL	18
**P3**	Pharmacy Practice Pharmacy Practice Lab Applied Pharmacokinetics * Disease State Management—II (DSM) * Pharmacy Management Research and Literature Evaluation—I Introductory Pharmacy Practice Experience—III (IPPE—III) Disease state Management—II (DSM)	3 1 3 5 3 1 1 0	Disease state Management—III (DSM) * Pharmacy Management/Pharmacoeconomics Pharmacy Law Research and Literature Evaluation—II * Basic and Clinical Nutrition Pharmacy Elective Introductory Pharmacy Practice Experience—III (IPPE—III) Disease state Management—II (DSM)	5 3 2 1 3 2 (3) 1 0
	TOTAL	17	TOTAL	17 (18)

* chr = credit hour.

**Table 2:** Characteristics of study sample (*n* = 384).

Characteristics	Professional Year			Total Sample (*n* = 384)
	P1 (*n* = 133)	P2 (*n* = 130)	P3 (*n* = 121)	
**Sociodemographic Factors**				
Age, Years Mean ± SD (Range)	24.0 ± 4.0 (19, 40)	25.5 ± 5.0 (20, 47)	25.1 ± 3.3 (21, 41)	24.8 ± 4.2 (19, 47)
Gender, Female %	65.4	70.8	63.6	66.7
Race/Ethnicity %				
Asian	1.5	0.0	2.5	1.3
Black/African American	38.4	53.9	44.6	45.6
White	24.8	12.3	15.7	17.7
Asian American	31.6	27.7	35.5	31.5
Others	3.8	6.2	1.7	3.9
Marital Status, Single %	90.2	86.9	87.6	88.3
Number of Children Mean ± SD (Range)	0.1 ± 0.6 (0, 4)	0.2 ± 0.7 (0, 3)	0.2 ± 0.5 (0, 3)	0.2 ± 0.6 (0, 4)
Proportion of Students with Children %	9.0	14.6	13.2	12.2
**Professional and Work-Related Activities**				
Pharmacy Board Hours Median (IQR)	0 (0, 100)	250 (100, 500)	500 (450, 500)	250 (0, 500)
Employment/Work Type %				
No Job	47.4	23.1	12.4	28.1
Chain Store	27.1	50.0	64.5	46.6
Hospital Retail	3.0	6.9	9.9	6.5
Hospital Pharmacy	0.0	0.0	0.0	0.0
Independent Pharmacy	2.3	5.4	9.1	5.5
Other	20.2	14.6	4.1	13.3
Member of Pharmacy Professional Organization, Yes %	65.4	73.9	88.4	75.5
Number of Pharmacy Professional Organization Mean ± SD (Range)	0.9 ± 0.7 (0, 3)	1.4 ± 1.0 (0, 3)	1.7 ± 1.1 (0, 4)	0.2 ± 0.6 (0, 4)
**Extracurricular Activities**				
Member of Nonpharmacy Professional Organization, Yes %	7.5	13.9	5.8	9.1
Officer in Nonpharmacy Professional Organization, %	0.0	1.5	4.1	1.8
**Academic Performance (GPA)**				
Fall GPA Mean ± SD (Range)	2.9 ± 0.6 (1.2, 4.0)	2.9 ± 0.6 (1.7, 4.0)	3.0 ± 0.5 (1.8, 4.0)	2.9 ± 0.6 (1.2, 4.0)
Spring GPA Mean ± SD (Range)	3.2 ± 0.5 (1.5, 4.0)	2.9 ± 0.6 (1.3, 4.0)	2.8 ± 0.5 (0.8, 4.0)	3.0 ± 0.6 (0.8, 4.0)

Note: SD: Standard Deviation; IQR: Interquartile Range; GPA: Grade Point Average; P1: Professional Year 1; P2: Professional Year 2; P3: professional Year 3.

**Table 3:** Association of workload with academic performance (grade point average).

**Fall Semester**				
**Work Measures**	**Model I** **β ± SE (*p*-Value)**	**Model II** **β ± SE (*p*-Value)**	**Model III** **β ± SE (*p*-Value)**	**Model IV** **β ± SE (*p*-Value)**
Pharmacy-Related Work	0.193 ± 0.110 (0.0809)	0.164 ± 0.110 (0.1381)	0.159 ± 0.107 (0.1382)	0.163 ± 0.107 (0.1284)
Nonpharmacy-Related Work	0.312 ± 0.123 (0.0114)	0.304 ± 0.122 (0.0133)	0.295 ± 0.118 (0.0046)	0.295 ± 0.118 (0.0131)
Work Hours per Week	−0.016 ± 0.005 (0.0016)	−0.014 ± 0.005 (0.0058)	−0.011 ± 0.005 (0.0236)	−0.011 ± 0.005 (0.0236)
**Spring Semester**				
**Work Measures**	**Model I** **β ± SE (*p*-Value)**	**Model II** **β ± SE (*p*-Value)**	**Model III** **β ± SE (*p*-Value)**	**Model IV** **β ± SE (*p*-Value)**
Pharmacy-Related Work	0.169 ± 0.111 (0.1308)	0.140 ± 0.111 (0.2026)	0.149 ± 0.107 (0.1635)	0.148 ± 0.107 (0.1696)
Nonpharmacy-Related Work	0.273 ± 0.122 (0.0258)	0.265 ± 0.121 (0.0295)	0.274 ± 0.117 (0.0201)	0.274 ± 0.117 (0.0202)
Work Hours per Week	−0.014 ± 0.005 (0.0113)	−0.011 ± 0.005 (0.0308)	−0.010 ± 0.005 (0.0537)	−0.010 ± 0.005 (0.0522)

Note: Model I: Relationship between the dependent variable (DV) and independent variable (IV) plus covariates—student classification and number of pharmacy board hours completed. Model II: Model I plus the demographic factors—gender, age, marital status, number of children, and race/ethnicity. Model III: Model II plus involvement in student professional organizations. Model IV: Model III plus involvement of fraternities or sororities (fully adjusted model).

## References

[1] Chisholm M.A., Cobb H.H., Dipro J.T. (1999). Development and validation of a model that predicts the academic ranking of first-year pharmacy students. Am. J. Pharm. Educ..

[2] McCall K.L., MacLaughlin E.J., Fike D.S., Ruiz B. (2007). Preadmission predictors of PharmD graduates’ performance on NAPLEX. Am. J. Pharm. Educ..

[3] Chisholm M.A., Cubb H.H., Kotzan J.A. (1995). Significant factors predicting academic success of first-year pharmacy students. Am. J. Pharm. Educ..

[4] Kuncel N.R., Crede M., Thomas L.L., Klieger D.M., Seiler S.N., Woo S.E. (2005). A meta-analysis of validity of pharmacy college admission test (PCAT) and grade predictors of pharmacy student performance. Am. J. Pharm. Educ..

[5] Sansgiry S.S., Bhosle M., Sail K. (2006). Factors that affect academic performance among pharmacy students. Am. J. Pharm. Educ..

[6] Schauner S., Hardinger K.L., Graham M.R., Garavalia L. (2013). Admission variables predictive of academic struggle in a PharmD program. Am. J. Pharm. Educ..

[7] McCall K.L., Allen D.D., Fike D.S. (2006). Predictors of academic success in a doctor of pharmacy program. Am. J. Pharm. Educ..

[8] Mar E., Barnett M.J., Tang T., Sasaki-Hill D., Kuperberg J.R., Knapp K. (2010). Impact of previous work experience on pharmacy school academic performance. Am. J. Pharm. Educ..

[9] Charupatanapong N., Mccornick W.C., Rascati K.L. (1994). Predicting academic performance of pharmacy students: Demographic comparisons. Am. J. Pharm. Educ..

[10] Webb C.T., Sadlacek W., Cohen D., Shields P., Gracely E., Hawkins M., Nieman L. (1979). The impact of nonacademic variables on performance at two medical schools. J. Nat. Med. Assoc..

[11] Rochford C., Connolly M., Drennan J. (2009). Paid part-time employment and academic performance of undergraduate nursing students. Nurs. Educ. Today.

[12] Nonis S.A., Hudson G.I. (2006). Academic performance of college students: Influence of time spent studying and working. J. Educ. Bus..

[13] García-Vargas M.C., Rizo-Baeza M., Cortés-Castell E. (2016). Impact of paid work on the academic performance of nursing students. PeerJ.

[14] Salamonson Y., Andrew S. (2006). Academic performance in nursing students: Influence of part-time employment, age and ethnicity. J. Adv. Nurs..

[15] Reyes H., Hartin V., Loftin C., Davenport D., Carter V. (2012). The impact of employment on nursing students’ academic performance. Nurse Educ..

[16] Chisholm-Burns M.A., Spivey C.A., Byrd D.C., McDonough S., Phelps S.J. (2017). Examining the Association Between the NAPLEX, Pre-NAPLEX, and Pre- and Post-admission Factors. Am. J. Pharm. Educ..

